# Is Cryoballoon Ablation Preferable to Radiofrequency Ablation for Treatment of Atrial Fibrillation by Pulmonary Vein Isolation? A Meta-Analysis

**DOI:** 10.1371/journal.pone.0090323

**Published:** 2014-02-28

**Authors:** Junxia Xu, Yingqun Huang, Hongbin Cai, Yue Qi, Nan Jia, Weifeng Shen, Jinxiu Lin, Feng Peng, Wenquan Niu

**Affiliations:** 1 Department of Geratology, Fuzhou General Hospital of Nanjing Command, PLA, Fuzhou, Fujian, China; 2 Department of Geratology, Fozhou General Hospital, Fujian Medical University, Fuzhou, Fujian, China; 3 Department of Cardiology, The First Affiliated Hospital of Fujian Medical University, Fuzhou, Fujian, China; 4 Department of Epidemiology, Capital Medical University Affiliated Beijing An Zhen Hospital, Beijing Institute of Heart, Lung and Blood Vessel Diseases, Beijing, China; 5 Department of Cardiology, The Fourth People’s Hospital of Shenzhen, Shenzhen, Guangdong, China; 6 Department of Cardiology, Ruijin Hospital, Shanghai Jiao Tong University School of Medicine, Shanghai, China; 7 Department of Human Genetics and Biostatistics, Institute of Cardiovascular Disease, Dalian Medical University, Dalian, Liaoning, China; 8 Center for Evidence-Based Medicine, Institute of Cardiovascular Disease, Dalian Medical University, Dalian, Liaoning, China; 9 State Key Laboratory of Medical Genomics, Ruijin Hospital, Shanghai Jiao Tong University School of Medicine, Shanghai, China; Medical University Innsbruck, Austria

## Abstract

**Objective:**

Currently radiofrequency and cryoballoon ablations are the two standard ablation systems used for catheter ablation of atrial fibrillation; however, there is no universal consensus on which ablation is the optimal choice. We therefore sought to undertake a meta-analysis with special emphases on comparing the efficacy and safety between cryoballoon and radiofrequency ablations by synthesizing published clinical trials.

**Methods and Results:**

Articles were identified by searching the MEDLINE and EMBASE databases before September 2013, by reviewing the bibliographies of eligible reports, and by consulting with experts in this field. Data were extracted independently and in duplicate. There were respectively 469 and 635 patients referred for cryoballoon and radiofrequency ablations from 14 qualified clinical trials. Overall analyses indicated that cryoballoon ablation significantly reduced fluoroscopic time and total procedure time by a weighted mean of 14.13 (95% confidence interval [95% CI]: 2.82 to 25.45; P = 0.014) minutes and 29.65 (95% CI: 8.54 to 50.77; P = 0.006) minutes compared with radiofrequency ablation, respectively, whereas ablation time in cryoballoon ablation was nonsignificantly elongated by a weighted mean of 11.66 (95% CI: −10.71 to 34.04; P = 0.307) minutes. Patients referred for cryoballoon ablation had a high yet nonsignificant success rate of catheter ablation compared with cryoballoon ablation (odds ratio; 95% CI; P: 1.34; 0.53 to 3.36; 0.538), and cryoballoon ablation was also found to be associated with the relatively low risk of having recurrent atrial fibrillation (0.75; 0.3 to 1.88; 0.538) and major complications (0.46; 0.11 to 1.83; 0.269). There was strong evidence of heterogeneity and low probability of publication bias.

**Conclusion:**

Our findings demonstrate greater improvement in fluoroscopic time and total procedure duration for atrial fibrillation patients referred for cryoballoon ablation than those for radiofrequency ablation.

## Introduction

Pulmonary vein isolation (PVI) via catheter ablation has become the recommended choice of treatment for patients with drug-refractory paroxysmal or persistent atrial fibrillation [1]. Conventionally radiofrequency is the preferred source of energy for ablation procedures, whereas its application has been limited by disrupting tissues due to excess heating or generation of inhomogeneous lesions [2], [3]. An alternative energy source, cryothermal energy, has recently been developed to overcome this limitation [4]. The cryoballoon catheter is composed of an inner and an outer balloon, and liquid nitrous oxide is delivered into the inner lumen of the balloon and changed into gas, thereby cooling the surrounding tissues to interrupt cellular metabolism and electrical activity. However, the potential benefits of cryoballoon ablation over radiofrequency ablation at present are still subject to an ongoing debate. For example, Linhart et al reported a similar success rate between cryoballoon and radiofrequency ablations [5], whereas the success rate for cryoballoon ablation was obviously high in a clinical trial by Kojodjojo et al [6]. It is worth noting that the majority of published trials on this topic are seriously underpowered, and most are even nonrandomized clinical trials. Given the accumulation of data, we therefore sought to undertake a meta-analysis of clinical trials that compared cryoballoon ablation with radiofrequency ablation in terms of the efficacy and safety for electrical isolation of pulmonary veins.

## Methods

This meta-analysis of clinical trials was carried out in accordance with the guidelines set forth by the Preferred Reporting Items for Systematic Reviews and Meta-analyses (PRISMA) statement (Supplementary Checklist S1) [7].

### Search strategy

Articles were identified by searching MEDLINE and EMBASE electronic databases from the earliest possible year to September 2013, by reviewing the bibliographies of original eligible reports, and by consulting with experts in this field. The key terms included ‘pulmonary vein’, ‘ablation’, ‘radiofrequency’, ‘cryothermal’, or ‘cryoballoon’, together with ‘atrial fibrillation’ or ‘arrhythmias’. Searching results were restricted to ‘clinical trials’ published in ‘English’ language.

### Trial selection

The titles and abstracts of 140 potentially relevant articles were evaluated independently by two investigators (F.P. and W.N.) and the full texts of 54 articles were obtained for further evaluation in duplicate. To avoid double counting of study patients, the corresponding authors were contacted for inquiries if necessary. For trials that produced more than one publication using the same study, data from the most recent or most complete publication were extracted.

### Inclusion/exclusion criteria

For inclusion, eligible trials should fulfill the following criteria (all must be satisfied): (1) to involve patients refractory to antiarrhythmic drugs and then referred for PVI by catheter ablation; (2) under treatment of either radiofrequency (including irrigated radiofrequency) or cryoballoon ablation for the first time; (3) to compare either of fluoroscopic time, total procedure time, ablation time, success rate of PVI, and the percentages of recurrent atrial fibrillation and major complications between cryoballoon or radiofrequency ablations. Trials were excluded (one was sufficient for exclusion) if they were cross-over trials or if they were conference abstracts, case reports, case series, editorials, review articles, or non-English articles.

### Data extraction

The primary outcome was the success rate of PVI, fluoroscopic time, total procedure time and ablation time. Secondary outcomes consisted of freedom from atrial fibrillation at the end of follow-up and major complications including cardiac tamponade, stroke or transient ischemic attack, pulmonary edema, phrenic nerve palsy, pulmonary vein stenosis, atrioesophageal fistula or death.

From each qualified article, two investigators (F.P. and W.N.) independently extracted the following data if available and entered them into a standard Excel template (Microsoft Corp, Redmond, WA): the first author’s surname, publication year, ethnicity, study design, the manufacturer and type of cryoballoon, radiofrequency type, matched information, sample size, fluoroscopic time, total procedure time, ablation time, success rate of PVI, recurrence of atrial fibrillation and major complications, as well as the characteristics of trial patients including age, gender, atrial fibrillation duration, left atrium diameter, previous percutaneous ablation, paroxysmal atrial fibrillation, left ventricular ejection fraction (LVEF), the percentages of coronary artery disease (CAD), hypertension and diabetes between the two arms.

Paroxysmal atrial fibrillation was defined as self-terminating episodes lasting <7 days, persistent atrial fibrillation as episodes between ≥7 days, or requiring a cardioversion to terminate. A distinction between persistent and long-lasting persistent atrial fibrillation was not made. The success of PVI was defined as complete PVI, which was confirmed by the disappearance of all pulmonary vein potentials or the dissociation of pulmonary vein potentials from left atrial activity.

Hypertension was diagnosed as the presence of elevated systolic (≥140 mmHg) and/or diastolic (≥90 mmHg) blood pressure, or current use of antihypertensive medications. Diabetes was defined as fasting plasma glucose levels ≥7.0 mmol/L or non-fasting plasma glucose levels ≥11.0 mmol/L, or taking hypoglycemic drugs or receiving parenteral insulin therapy. Data were compared and disagreements regarding whether to include or exclude a trial were resolved by consensus between all authors.

### Statistical analysis

Quantitative outcomes were compared by weighted mean difference (WMD) and its 95% confidence interval (95% CI) between cryoballoon and radiofrequency ablation procedures. Categorical variables were evaluated by weighted odds ratio (OR) and the corresponding 95% CI, which were calculated by the Mantel-Haenszel method. The pooled effect estimates were calculated using the inverse-variance weighting under both fixed-effects and DerSimonian & Laird [8] random-effects models.

Heterogeneity was assessed by χ^2^ test and quantified using the inconsistency index (*I* ^2^) statistic, which ranges from 0% to 100% and is defined as the percentage of the observed between-trial variability that is due to heterogeneity rather than chance. Given that the fixed- and random-effects models produced similar results in the absence of heterogeneity, the random-effects model is thereby adopted.

Sensitivity analysis was performed to assess the contribution of each individual trial to pooled effect estimate by sequentially removing each trial in turn. Meta-regression analysis was used to evaluate the extent to which different trial-level variables explained the heterogeneity of effect estimates between cryoballoon and radiofrequency ablation procedures.

Publication bias was assessed by the Begg’s and Egger’s tests. The trim-and-fill method was adopted to estimate the number and outcomes of potentially missing trials due to publication bias. P<0.05 was considered statistically significant except for the *I* ^2^, Begg’s and Egger’s statistics where a significance level was set as P<0.10 [9]. The statistical analyses described above were completed using the STATA software (StataCorp, College Station, TX, version 11.2 for Windows).

## Results

### Eligible trials

**Figure 1:**
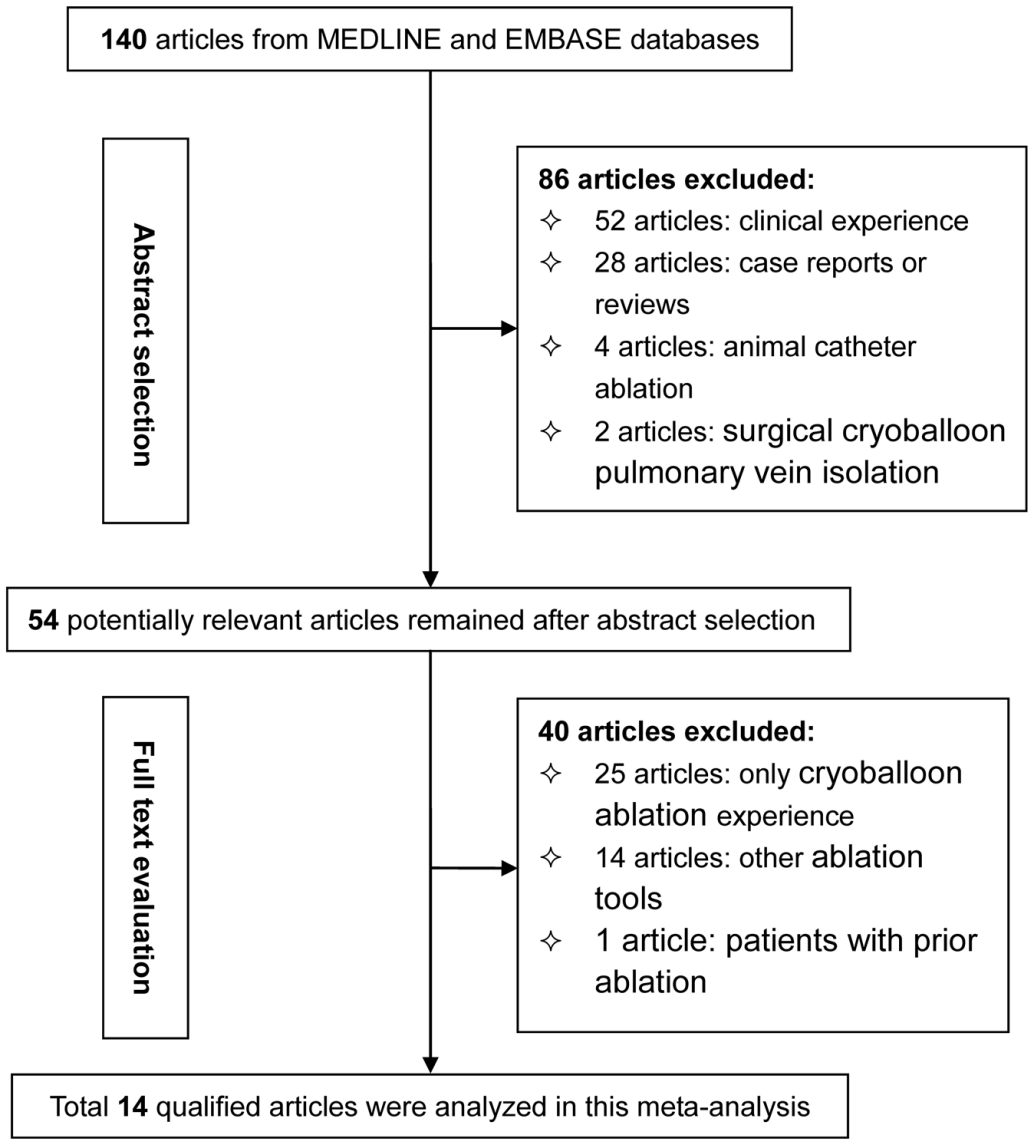
Flow diagram of search strategy and study selection.

Baseline characteristics of all study patients and a flow diagram schematizing the process of excluding articles with specific reasons are summarized in Tables 1 and 2 and Figure 1, respectively. Of 140 potentially relevant articles identified in initial literature search, 14 qualified articles involving 1104 patients referred for catheter ablation were analyzed [5], [6], .

**Table 1:** Baseline characteristics of study patients of all qualified trials in this meta-analysis.

Author (year)	Country	Cryo type	Manufacturer	RF type	Design	Matched	Number	Age (yrs)	Gender (Males)
Linhart M et al (2009)	Germany	23 or 28 mm	Arctic Front	Irrigated RF	Nonrandomized	age, sex	20/20	59.9/58.5	0.75/0.75
Sauren LD et al (2009)	Netherlands	28 mm	Arctic Front	Irrigated RF	Nonrandomized	NA	10/10	58/53	0.7/1
Chierchia GB et al (2010)	Belgium	28 mm	Arctic Front	Irrigated RF	Nonrandomized	NA	46/87	56/56	0.78/0.79
Kojodjojo P et al (2010)	UK	28 mm	Arctic Front	Irrigated RF	Nonrandomized	NA	90/53	57.3/59.3	0.75/0.77
Kuhne M et al (2010)	Switzerland	28 mm	NA	Irrigated RF	Nonrandomized	age, sex	18/25	58/59	0.88/0.84
Sorgente A et al (2010)	Belgium	28 mm	Arctic Front	Irrigated RF	Nonrandomized	NA	30/29	56/56.1	0.74/0.9
Gaita F et al (2011)	Italy	23 or 28 mm	Arctic Front	Irrigated RF	Nonrandomized	NA	36/36	55/57	0.69/0.67
Herrera SC et al (2011)	Germany	23 or 28 mm	Arctic Front	Irrigated RF	Nonrandomized	NA	23/27	61/61	0.65/0.74
Neumann T et al (2011)	Germany	NA	Arctic Front	Irrigated RF	Nonrandomized	NA	45/44	56/58	0.53/0.73
Herrera SC et al (2012)	Germany	23 or 28 mm	Arctic Front	Irrigated RF	Randomized	NA	30/30	57/56	0.83/0.77
Schmidt M et al (2012)	Germany	23 or 28 mm	Arctic Front	Irrigated RF	Nonrandomized	NA	37/178	60/63	0.76/0.84
Betts TR et al (2013)	UK	28 mm	Arctic Front	Irrigated RF	Nonrandomized	age, sex	21/21	54/55	0.67/0.81
Maagh P et al (2013)	Germany	28 mm	Arctic Front	Irrigated RF	Nonrandomized	NA	30/42	59.9/60.6	0.633/0.69
Schmidt B et al (2013)	Germany	28 mm	NA	Irrigated RF	Randomized	NA	33/33	66/63	NA/NA

*Abbreviations:* Cryo type, type of cryoballoon; RF type, type of radiofrequency ablation; NA, not available. Digital data were expressed as counting or percentages between cryoballoon/radiofrequency techniques unless otherwise indicated.

**Table 2:** Baseline characteristics of study patients of all qualified trials in this meta-analysis.

Author (year)	AF-d (yrs)	LA-d (mm)	PAF	LVEF (%)	CAD	Hypertens	Diabetes	Success rate	Recurrence rate	Complications
Linhart M et al (2009)	7/7	NA/NA	1/1	59.5/62.5	0.1/0	0.6/0.25	0/0.05	0.5/0.45	0.5/0.55	NA/NA
Sauren LD et al (2009)	NA/NA	NA/NA	1/0.9	NA/NA	NA/NA	NA/NA	NA/NA	NA/NA	NA/NA	NA/NA
Chierchia GB et al (2010)	3.3/3.2	41/42	NA/NA	64/64	0.086/0.05	0.24/0.23	NA/NA	NA/NA	NA/NA	NA/NA
Kojodjojo P et al (2010)	5.6/6	39.6/41.6	1/1	65/60.3	0.06/0.06	0.47/0.26	NA/NA	0.79/0.42	0.21/0.58	NA/NA
Kuhne M et al (2010)	5/3.25	41/42	1/1	60/58	0.16/0.16	NA/NA	NA/NA	NA/NA	NA/NA	NA/NA
Sorgente A et al (2010)	2.8/3.4	40.8/42.4	0.89/0.69	63.9/64.2	0.11/0.07	0.29/0.59	0/0.03	0.66/0.66	0.34/0.35	NA/NA
Gaita F et al (2011)	5.08/6.66	41/43	NA/NA	63/64	NA/NA	0.36/0.31	NA/NA	NA/NA	NA/NA	NA/NA
Herrera SC et al (2011)	NA/NA	40/42	0.65/0.48	NA/NA	NA/NA	0.61/0.59	NA/NA	NA/NA	NA/NA	0.96/0.93
Neumann T et al (2011)	NA/NA	51/53	1/0.614	62/58	0.13/0.07	0.51/0.59	0/0.09	NA/NA	NA/NA	NA/NA
Herrera SC et al (2012)	4.2/5.6	41.4/40	0.7/0.567	NA/NA	NA/NA	0.43/0.47	NA/NA	0.63/0.8	0.37/0.2	0.867/1
Schmidt M et al (2012)	0.83/0.92	46/46	1/0.54	60/58	0.2/0.2	0.58/0.61	0.13/0.11	NA/NA	NA/NA	0.95/0.98
Betts TR et al (2013)	NA/NA	42/45	0.67/0.48	NA/NA	NA/NA	NA/NA	NA/NA	NA/NA	NA/NA	NA/NA
Maagh P et al (2013)	1.04/0.64	38.9/37.5	0.7/0.64	NA/NA	0.13/0.17	0.2/0.095	NA/NA	0.73/0.72	0.27/0.28	NA/NA
Schmidt B et al (2013)	NA/NA	40/41	NA/NA	59/58	0.21/0.18	0.76/0.7	0.06/0.06	NA/NA	NA/NA	1/1

*Abbreviations:* AF-d, atrial fibrillation duration; LA-d, left atrium diameter; PAF, paroxysmal atrial fibrillation; LVEF, left ventricular ejection fraction; CAD, coronary artery disease; Hypertens, hypertension; NA, not available. Digital data were expressed as counting or percentages between cryoballoon/radiofrequency techniques unless otherwise indicated.

All clinical trials were conducted in Caucasians from European countries and published between 2009 and 2013. Eight of 14 trials adopted 28 mm cryoballoon [6], [10−13], , and five adopted mixed cryoballoon of 23 mm and 28 mm [5], [14], [15], [17], [18]. All trials adopted the irrigated radiofrequency. All but two trials with missing information [12], [21] used cryoablation catheter from the Arctic Front (Medtronic, USA). Two of 14 trials had a randomized study design [17], [21], and three trials had patients matched on age and gender between cryoballoon and radiofrequency ablations [5], [12], [19].

There were respectively 469 and 635 patients referred for cryoballoon and radiofrequency ablation procedures in PVI for the treatment of atrial fibrillation. Distributions of age, atrial fibrillation duration, LVEF, previous percutaneous ablation, CAD, hypertension and diabetes were comparable between patients referred for cryoballoon and radiofrequency ablations (P>0.05). There were more males for radiofrequency ablation (79.2%) than cryoballoon ablation (72.0%) (P = 0.0284). Left atrium diameter was slightly elevated for radiofrequency ablation (42.96% versus 41.89% for cryoballoon ablation, P = 0.0212). By contrast, there were more patients with paroxysmal atrial fibrillation referred for cryoballoon ablation (87.36%) than radiofrequency ablation (71.91%) (P = 0.0076).

### Efficacy

**Figure 2:**
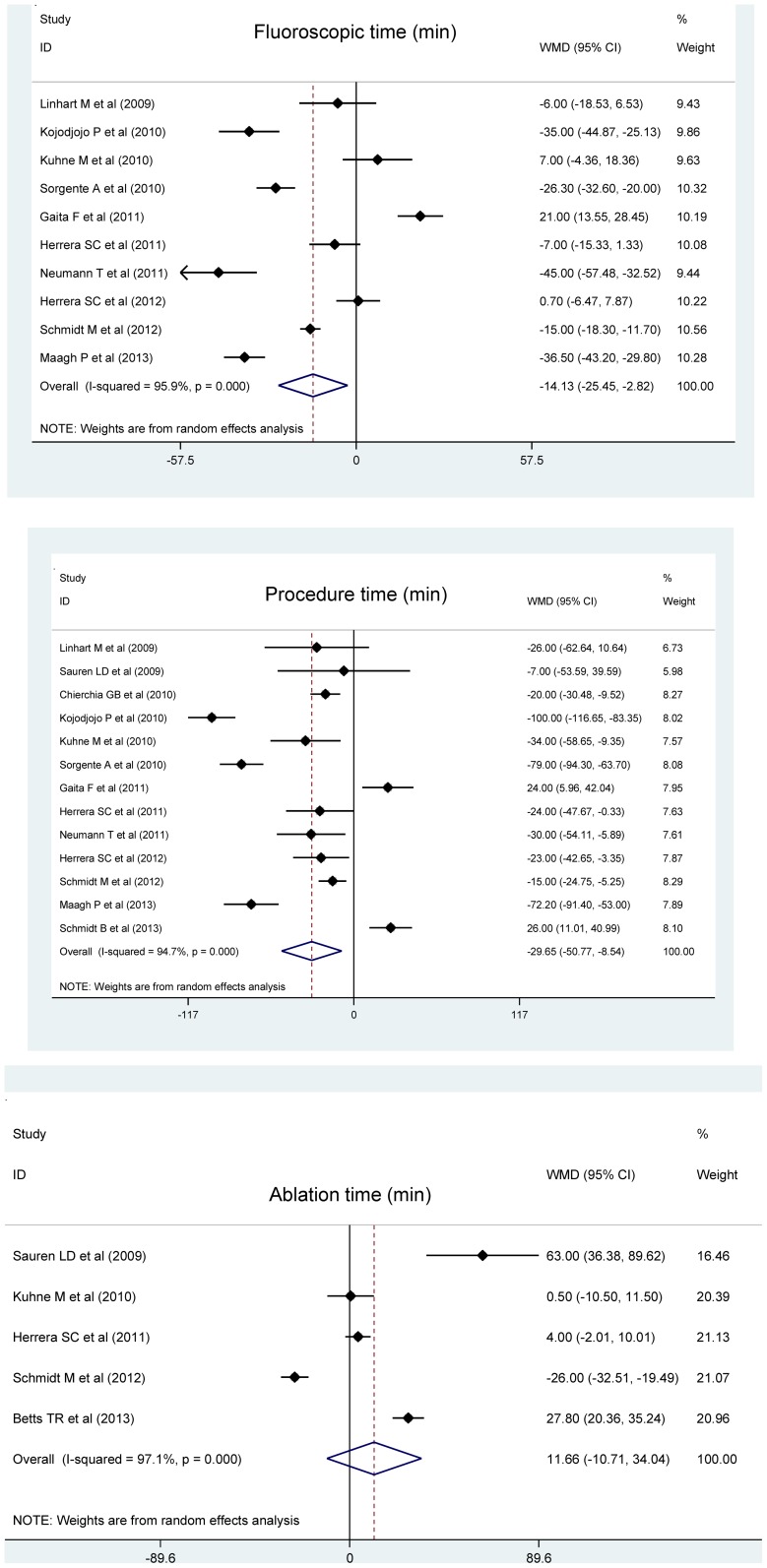
Forest plots of changes of fluoroscopy time, total procedure time and ablation time for cryoballoon ablation versus radiofrequency ablation.

Pooling the results of all qualified trials observed that cryoballoon ablation significantly reduced fluoroscopic time and total procedure time by a weighted mean of 14.13 (95% confidence interval [95% CI]: 2.82 to 25.45; P = 0.014) minutes and 29.65 (95% CI: 8.54 to 50.77; P = 0.006) minutes compared with radiofrequency ablation, respectively (Figure 2). In contrast, cryoballoon ablation had longer yet nonsignificant ablation time than radiofrequency ablation (WMD = 11.66 minutes; 95% CI: −10.71 to 34.04; P = 0.307). It is worth noting that the wide confidence intervals generated might result from the small sample sizes of clinical trials involved and the sharply divergent results of the very few trials from overall estimates.

**Figure 3:**
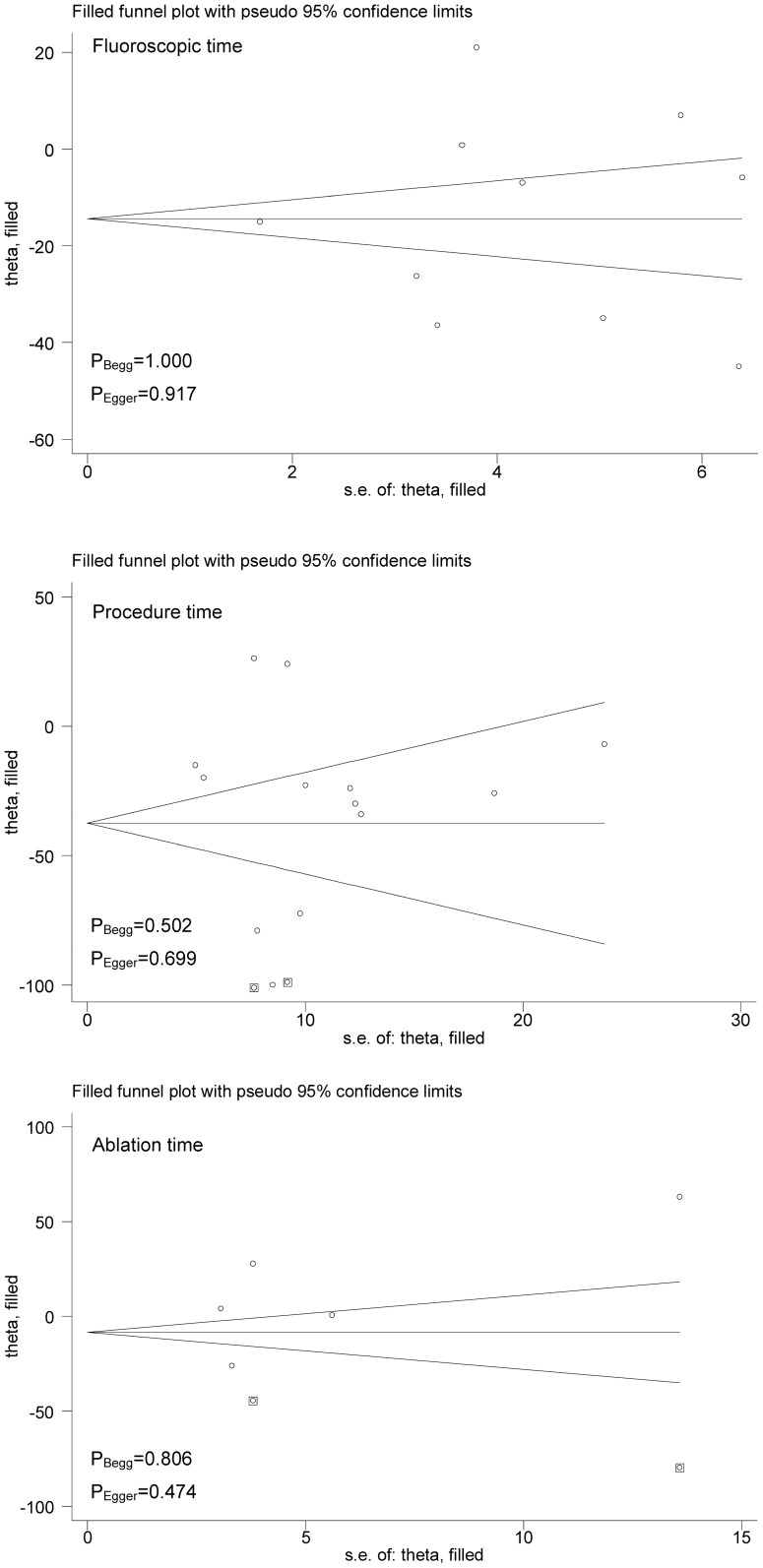
Trim-and-fill funnel plots of fluoroscopy time, total procedure time and ablation time for cryoballoon ablation versus radiofrequency ablation.

The *I* ^2^ values, which quantified heterogeneity between trails, were 95.9%, 94.7% and 97.1% for fluoroscopic time, total procedure time and ablation time, respectively, suggesting strong evidence of between-trial heterogeneity (all P<0.001). As reflected by the Begg’s and Egger’s tests (Figure 3), there were low probabilities of publication bias for all comparisons. Further adopting the trim-and-fill adjustment method yielded no material changes in pooled effects estimates, and as estimated only two missing trials were required for ablation time to make the funnel plot symmetrical (Figure 3).

### Success rate

**Figure 4:**
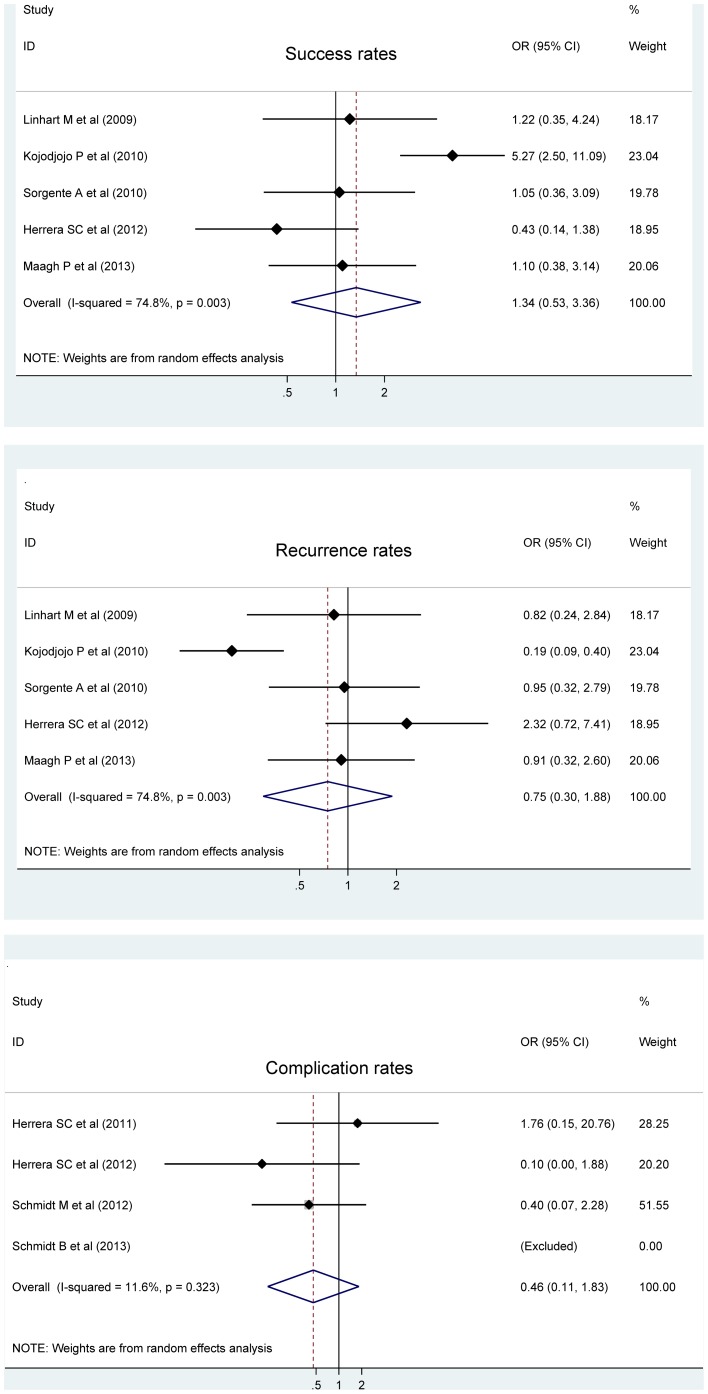
Forest plots of success rate, recurrence of atrial fibrillation, complication rate for cryoballoon ablation versus radiofrequency ablation.

Success rate of catheter ablation was relatively higher in patients referred for cryoballoon ablation than radiofrequency ablation, the difference exhibiting no statistical significance (OR; 95% CI; P: 1.34; 0.53 to 3.36; 0.538) (Figure 4). There was evident heterogeneity (*I* ^2^ = 74.8%; P = 0.003) and no publication bias (Figure S1).

### Recurrence and complications

Cryoballoon ablation was also found to be associated with relatively low risk of having recurrent atrial fibrillation (0.75; 0.3 to 1.88; 0.538) and major complications (0.46; 0.11 to 1.83; 0.269) (Figure 4). As indicated by the *I* ^2^ statistic, heterogeneity was significant for recurrence rate (*I* ^2^ = 74.8%; P = 0.003) but not for complication rate (*I* ^2^ = 11.6%; P = 0.323).

### Sensitivity analysis

There was not an individual trial influencing the overall effect estimate significantly for all examined comparisons. After removing each trial and calculating the overall estimate for the remaining trials, the significance of the WMD or OR remained materially unchanged (Figure S2).

### Meta-regression analysis

To explore the extent to which trial-level variables account for heterogeneity, a panel of meta-regression analyses were conducted. None of examined variables including age, gender, study design, type of cryoballoon, matched information on age and gender, atrial fibrillation duration, left atrium diameter, previous percutaneous ablation, paroxysmal atrial fibrillation, LVEF, CAD, hypertension and diabetes, contributed significantly to the variation of effect estimates between cryoballoon and radiofrequency ablation procedures (P<0.05 for all) (data not shown).

## Discussion

The most noteworthy of this study was that there was greater improvement in fluoroscopic time and total procedure duration in patients referred for cryoballoon ablation than those for radiofrequency ablation in PVI of atrial fibrillation. Moreover, success rate of PVI, the percentages of recurrence of atrial fibrillation and major complications were comparable between the two procedures. To our knowledge, this is so far the first comprehensive meta-analysis comparing cryoballoon ablation with radiofrequency ablation in terms of the efficacy and safety for electrical isolation of pulmonary veins.

Currently radiofrequency and cryoballoon ablations are the two standard ablation systems used for catheter ablation of atrial fibrillation. As an alternative approach to conventional radiofrequency ablation, cryoballoon ablation has been recently developed for PVI. From a technologic viewpoint, a closer match between the cryoballoon size and the size of pulmonary vein ostium would allow for better balloon occlusion, which in turn produces more effective lesions [22]. It has been demonstrated that the catheter point-by-point cryoballoon is an effective approach to generate PVI with clinically satisfactory consequences [23]. Even more remarkably, compared with the first generation cryoballoon that was widely adopted in the majority of included trials in this meta-analysis, the second generation cryoballoon equipped with a modified refrigerant injection system has recently been introduced, and this novel balloon can provide a more homogeneous and effective cooling [24]. There is also evidence that the learning curve for cryoballoon ablation was much shorter than for radiofrequency ablation [25]. All these favorite characteristics will endow cryoballoon ablation with higher procedure efficiency and long-term success rate after cryoballoon ablation.

As reflected in our overall analyses, fluoroscopic time and total procedure duration were greatly improved by using cryoballoon ablation compared with radiofrequency ablation, consistent with the trends of most clinical trials [6], [16], [18], [20]. Contrastingly, there was longer ablation time in cryoballoon technique in this meta-analysis, likely due to the need for pre-procedural computerized tomography imaging, which can further increase the cumulative radiation dose received by the patients and overall costs. Although the success rate of catheter ablation was higher in patients referred for cryoballoon ablation than radiofrequency ablation, there was no observable statistical difference, possibly due to methodological limitations, including inadequate sample size, patient section, and lack of adjustment for confounders. Here, we cannot overlook the fact that in some clinical centers, selection of cryoballoon or radiofrequency ablation is largely based on the patient’s anatomy, that is, patients with unfavorable anatomy on computed tomography may be referred for radiofrequency ablation rather than cryoballoon ablation [22]. Moreover in clinical routine, the patients with high-risk features were more likely to have undergone the procedure with radiofrequency relative to cryoballoon ablation. Nevertheless, we believe that with the accumulation of operational experience, cryoballoon ablation’s advantage over radiofrequency ablation will become more and more obvious in clinical practice.

However, a note of caution should be added because heterogeneity might potentially limit the interpretation of our pooled effect estimates. In this meta-analysis, to account for the potential sources of heterogeneity between trials, we undertook a penal of meta-regression analyses, whereas we failed to identify any contributory confounders. The meta-regression analysis, albeit enabling both categorical and continuous variables to be considered, by itself does not have the methodological rigor of a properly designed study that is intended to test the effect of these confounders formally. On the other hand, we must recognize that our meta-regression analysis involved limited trials of insufficient sample sizes, rendering it incapable of performing subgroup analyses and detecting a small or moderate effect estimate. Our results, therefore, might underestimate the virtual changes between cryoballoon and radiofrequency ablations, and definitively there is a need for further large, randomized clinical trials to confirm or refute our findings.

Despite the clear strengths of this meta-analysis including low probabilities of publication bias and the robustness of statistical analyses, interpretation of our findings, however, should be viewed in light of several limitations. First, only two of 14 qualified trials were performed in a randomized design, raising the potential existence of potential biases and/or unmeasured confounders. Although randomized trials can minimize bias and are regarded as the gold standard for quantifying effect estimates, they may not be reflective of patients treated in general clinical practice [26]. Second, our total sample size of 1104 patients was not large enough to draw a firm conclusion, and there were more patients referred for radiofrequency ablation relative to cryoballoon ablation. Third, the left atrium size and percentage of paroxysmal atrial fibrillation were not proportional between the two ablation procedures in this meta-analysis, which might bias our findings, however, our further meta-regression analyses failed to detect their contributory influence on the effect estimates. Fourth, data on major complications were limited in this meta-analysis, and some complications such as phrenic nerve paralysis are typical complications in cryoballoon ablation but rare in radiofrequency ablation. Fifth, the fact that study patients were all Caucasians from European countries limited the generalizability of our findings, reinforcing the future validation in other ethnics. Last but not the least, as with all meta-analyses, despite a low probability of publication bias in this meta-analysis, selection bias cannot be completely excluded, since we merely identified articles from the English journals and published trials.

In conclusion, this study confirms and extends the findings of most clinical trials by demonstrating greater improvement in fluoroscopic time and total procedure duration in atrial fibrillation patients referred for cryoballoon ablation relative to those referred for radiofrequency ablation in PVI. However, it should be noted that success rate of PVI, the percentages of recurrent atrial fibrillation and major complications were comparable between the two procedures. For practical reasons, with the accumulation of data from large randomized clinical trials, successful validation of the present results will revolutionize the current clinical practice and healthcare system by bringing great benefits to doctors and patients alike in the near future.

## Supporting Information

Figure S1 **Trim-and-fill funnel plot of the success rate of pulmonary vein isolation for cryoballoon ablation versus radiofrequency ablation.**
(PDF)

Figure S2 **Sensitivity analyses of fluoroscopic time (A), total procedure time (B), ablation time (C) and success rate of pulmonary vein isolation (D) for cryoballoon ablation versus radiofrequency ablation.**
(PDF)

Checklist S1 **The PRISMA Checklist.**
(DOC)
