# Supplementary material for: Is Cryoballoon Ablation Preferable to Radiofrequency Ablation for Treatment of Atrial Fibrillation by Pulmonary Vein Isolation? A Meta-Analysis
Source: PLoS One. 2014 Feb 28;9(2):e90323. doi: 10.1371/journal.pone.0090323 (PMC3938670; doi:10.1371/journal.pone.0090323)

Supplementary Figure S1. Trim-and-fill funnel plot of the success rate of pulmonary vein isolation for cryoballoon ablation versus radiofrequency ablation

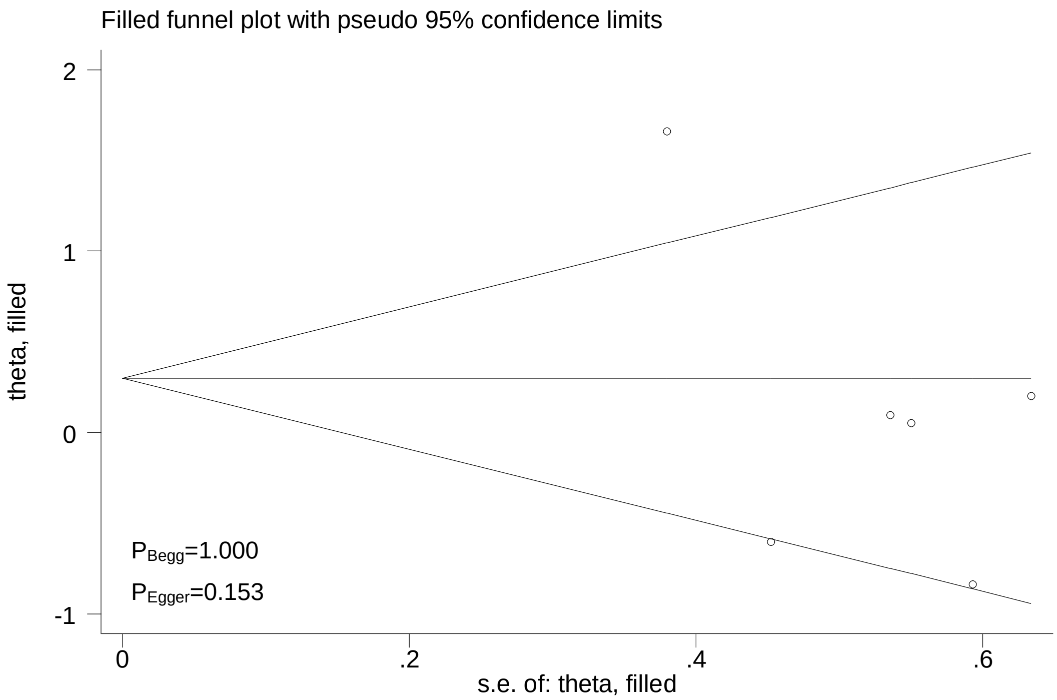

Supplement: Figure S1 — Trim-and-fill funnel plot of the success rate of pulmonary vein isolation for cryoballoon ablation versus radiofrequency ablation. (PDF) [file pone.0090323.s001.pdf]
