# Supplementary material for: Is Cryoballoon Ablation Preferable to Radiofrequency Ablation for Treatment of Atrial Fibrillation by Pulmonary Vein Isolation? A Meta-Analysis
Source: PLoS One. 2014 Feb 28;9(2):e90323. doi: 10.1371/journal.pone.0090323 (PMC3938670; doi:10.1371/journal.pone.0090323)

Supplementary Figure S2. Sensitivity analyses of fluoroscopic time (A), total procedure time (B), ablation time (C) and success rate of pulmonary vein isolation (D) for cryoballoon ablation versus radiofrequency ablation

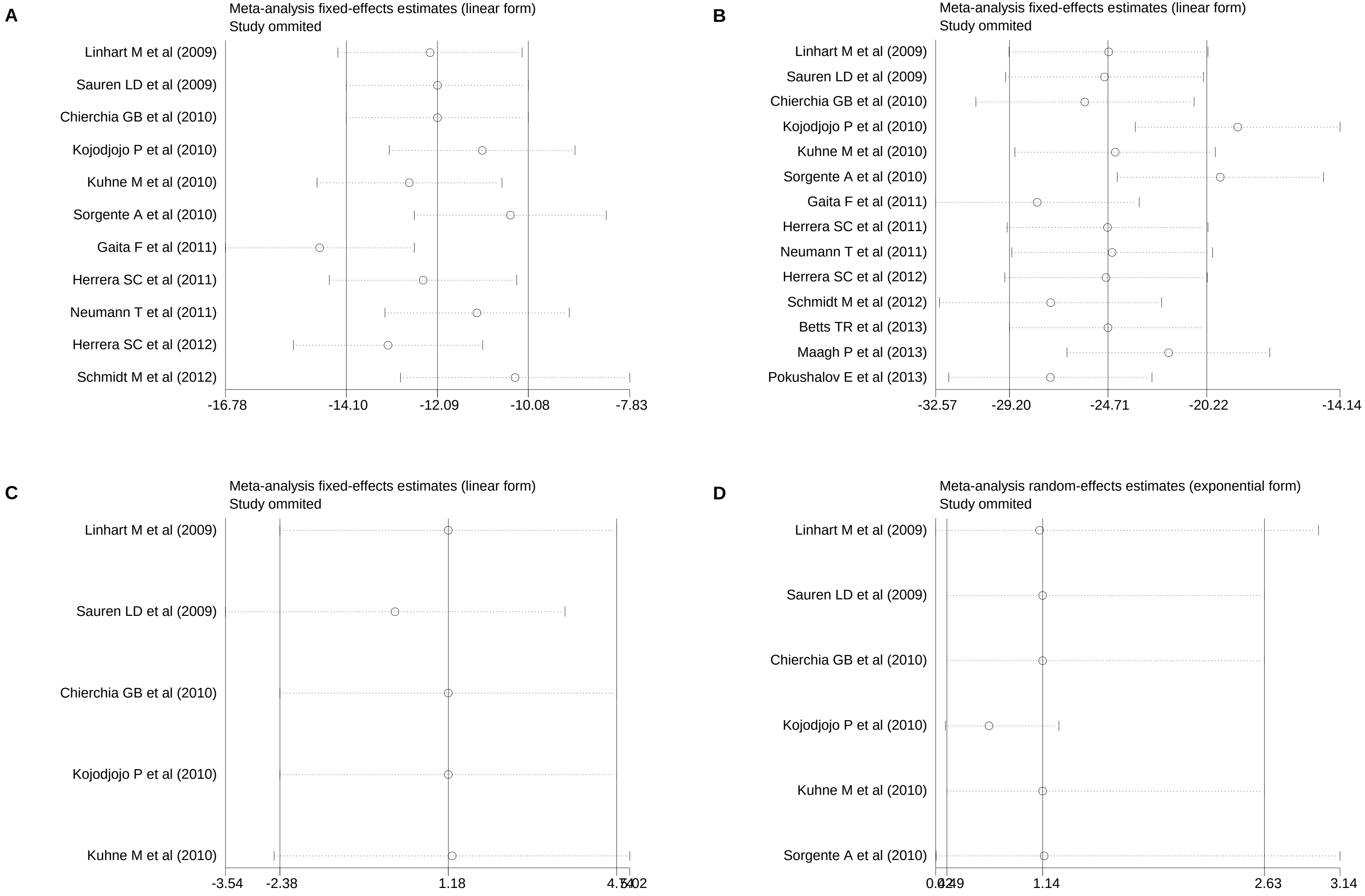

Supplement: Figure S2 — Sensitivity analyses of fluoroscopic time (A), total procedure time (B), ablation time (C) and success rate of pulmonary vein isolation (D) for cryoballoon ablation versus radiofrequency ablation. (PDF) [file pone.0090323.s002.pdf]
